# Archaeal and Bacterial Communities Associated with the Surface Mucus of Caribbean Corals Differ in Their Degree of Host Specificity and Community Turnover Over Reefs

**DOI:** 10.1371/journal.pone.0144702

**Published:** 2016-01-20

**Authors:** Pedro R. Frade, Katharina Roll, Kristin Bergauer, Gerhard J. Herndl

**Affiliations:** 1 Department of Limnology and Bio-Oceanography, Center of Ecology, University of Vienna, Althanstrasse 14, 1090 Vienna, Austria; 2 Caribbean Research and Management of Biodiversity (CARMABI) Foundation, Piscaderabaai z/n, PO Box 2090, Willemstad, Curaçao; 3 Department of Biological Oceanography, Royal Netherlands Institute for Sea Research (NIOZ), 1790AB Den Burg, The Netherlands; Wilfrid Laurier University, CANADA

## Abstract

Comparative studies on the distribution of archaeal *versus* bacterial communities associated with the surface mucus layer of corals have rarely taken place. It has therefore remained enigmatic whether mucus-associated archaeal and bacterial communities exhibit a similar specificity towards coral hosts and whether they vary in the same fashion over spatial gradients and between reef locations. We used microbial community profiling (terminal-restriction fragment length polymorphism, T-RFLP) and clone library sequencing of the 16S rRNA gene to compare the diversity and community structure of dominant archaeal and bacterial communities associating with the mucus of three common reef-building coral species (*Porites astreoides*, *Siderastrea siderea* and *Orbicella annularis*) over different spatial scales on a Caribbean fringing reef. Sampling locations included three reef sites, three reef patches within each site and two depths. Reference sediment samples and ambient water were also taken for each of the 18 sampling locations resulting in a total of 239 samples. While only 41% of the bacterial operational taxonomic units (OTUs) characterized by T-RFLP were shared between mucus and the ambient water or sediment, for archaeal OTUs this percentage was 2-fold higher (78%). About half of the mucus-associated OTUs (44% and 58% of bacterial and archaeal OTUs, respectively) were shared between the three coral species. Our multivariate statistical analysis (ANOSIM, PERMANOVA and CCA) showed that while the bacterial community composition was determined by habitat (mucus, sediment or seawater), host coral species, location and spatial distance, the archaeal community composition was solely determined by the habitat. This study highlights that mucus-associated archaeal and bacterial communities differ in their degree of community turnover over reefs and in their host-specificity.

## Introduction

Tropical reef-building corals are associated with dynamic and highly diverse consortia of microorganisms comprising Bacteria, Archaea, fungi, viruses and protists including the endosymbiotic algae *Symbiodinium* [1, 2]. At the interface between the coral host epithelium and the surrounding seawater, coral surface mucus forms a microbial biofilm acting as a defence barrier against a wide range of environmental stressors (reviewed in [3]). The phylogenetic diversity of microbial communities associated with corals is known to vary depending on surrounding environmental conditions [4], as well as on host interactions [5]. Studies on the diversity of coral-associated Bacteria using 16S rDNA have revealed a high diversity of bacterial ribotypes [1, 6] and studies on their archaeal counterparts suggested a similarly high diversity [2, 7]. Functionally, coral-associated microbes are crucial to the physiology of their hosts by contributing to pathogen defence and resistance and to biogeochemical cycling [8, 9]. Coral-associated microbes have been implicated in nitrogen fixation [10], putative ammonia oxidation [11] or the cycling of sulphur compounds [5]. Recent studies suggest that there is considerable variation in the composition and function of microbial assemblages associating with distinct habitats within a single reef location [12], and also among distinct coral compartments, such as mucus, tissue and skeleton [13]. A study on the coral core microbiome has recently identified rare bacterial taxa as putative endosymbionts likely contributing to the success of the dinoflagellate endosymbiosis in corals [14].

The spatial distribution of microbial populations and their dynamics in coral mucus are still poorly understood. The availability of nutrients provided by the coral host and/or its associated algal symbionts is probably an important structuring element for the partitioning of bacterial niches at the microscale [15, 16]. Also, there is spatial heterogeneity of bacterial communities even at the microscale within the mucus of individual colonies [17]. Previous studies investigating the biogeography of coral-associated microbial assemblages have found confounding patterns. While some studies indicated that host species-specificity of coral-associated microbes is consistent across large geographical scales [1], others have shown the opposite trend, with microbial assemblages composed of different lineages in different geographical locations but being similar in corals living in sympatry [18, 19]. A tentatively unifying view is that while holobiont macroorganisms determine the composition of their core microbiome, the microbial metabolism can vary depending on local conditions [4].

At present it is unclear whether archaeal and bacterial communities are identically structured over coral reefs or whether they differ in their host-specificity and variation in community structure across geographical scales. The current perception is that while bacterial assemblages associated with surface mucus show host species-specificity and phylogenetic variation across large spatial scales [20], Archaea do not establish species-specific associations [2, 7] and are rather uniform across large geographical scales [11]. However, definite studies investigating both archaeal and bacterial communities across the same geographical area and using the same set of samples are not available thus far. Comparing the variation in community structure of distinct organisms over the same geographical scale is indicative of their degree of response to environmental heterogeneity and spatial distance [21] and to the underlying processes of selection, drift, dispersal and mutation [22]. Although it is not the objective of the current study to resolve the specific contribution of each of these processes to the structuring of bacterial and archaeal communities, we pursue to find contrasting patterns between these domains of life regarding their host-specificity and their community turnover over spatial gradients [23].

The coastal waters off Curaçao and its reefs are poorly described regarding their microbial diversity and community structure. The few studies on coral-associated microbial communities in the reefs off Curaçao indicate that bacterial communities differ between coral hosts [24] and that conspecific corals located just a few meters apart can harbor significantly different bacterial assemblages [25]. In the present study, we aimed at obtaining insight into the environmental *versus* host control of prokaryotic communities associated with coral surface mucus by comparing the diversity and community structure of dominant bacterial and archaeal assemblages inhabiting the surface mucus of three distinct coral species across a spatial gradient ranging from meters to kilometers. We found substantial discrepancies between bacterial and archaeal communities associating with coral mucus, particularly regarding patterns of host-specificity and community turnover over a spatial gradient comprising distinct reef sites.

## Materials and Methods

### Study sites and sampling approach

**Fig 1:**
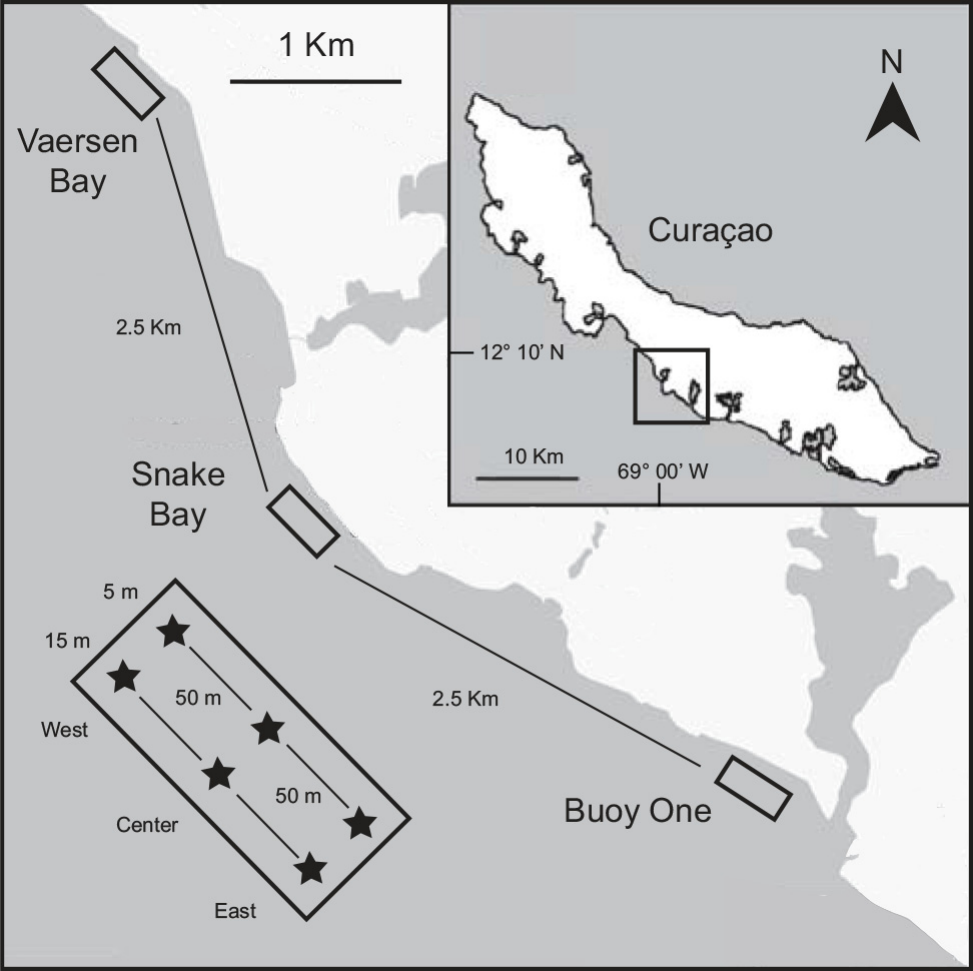
Study sites and sampling approach. Map of Curaçao with the three reef sites: Vaersen Bay, Snake Bay and Buoy One (upper panel). Lower left inset depicts the patch sampling approach used, with each site being sampled over three patches (West, Center and East) spanning two depths (5 and 15 m depth). Modified after Frade et al. [27].

Fieldwork took place on the island of Curaçao, southern Caribbean (Fig 1) based at CARMABI Foundation from 15–21 July 2011. Research on Curaçao was performed under the annual research permit (48584) issued by the Curaçaoan Ministry of Health, Environment and Nature (GMN) to the CARMABI Foundation. Three sampling sites were visited: Buoy One reef, Snake Bay and Vaersen Bay, located ca. 2.5 km apart from each other along the pollution gradient generated from the urban area of Willemstad and maintained by the predominant east to west current [26]. At each site, two depths (5 and 15m) were sampled with three reef patches per site and depth (named West, Centre and East), each located 50 m apart from the other one (see Fig 1). This sampling approach allowed us comparing neighboring reefs or reef patches located only tens of meters apart from each other within the same reef system.

At each of the 18 site-depth-patch sampling points, the mucus of three main Caribbean reef-building coral species, *Porites astreoides* (Poritidae), *Siderastrea siderea* (Siderastreidae) and *Orbicella annularis* (Faviidae), was sampled with a minimum of three colonies per species. Only healthy, light-exposed coral colonies were included in the study and only the uppermost surface of the colony was sampled. Mucus was collected during SCUBA diving by gently rolling sterile cotton swabs over the surface (ca. 9 cm^2^) of the coral colonies. Swabs were kept in 2 mL tubes with minimal seawater intrusion. Tubes were transported back to the lab in the dark at *in situ* temperature, flash-frozen in liquid nitrogen and kept at -80°C until further processing. Reference sediment samples (one per sampling patch; n = 18) and ambient water (one per depth for each site; n = 6) were taken from the vicinity of the sampled corals. Sediment was collected from the superficial layer with a 2 mL tube, while 2 L of ambient water was collected in plexiglas cylinders and in the laboratory immediately filtered onto a 0.2 μm polycarbonate filter (GTTP, Millipore). A total of 239 samples was taken.

### Nucleic acid extraction

DNA was extracted from all samples using the FastDNA Spin Kit for Soil (MP Biomedicals). Cells were mechanically lysed with FastPrep at a speed of 6.0 beats sec^-1^ for 40 sec. All DNA was eluted in 80 μL of DNase/pyrogen-free water. Based on quantifications of a single-copy housekeeping gene performed on similarly processed samples [27], we estimated that per sample between 10^4^−10^5^ cells were extracted from the mucus swabs, 10^6^−10^7^ cells from the sediment and 10^7^−10^8^ cells from the ambient water samples.

### Cloning, sequencing and phylogenetic analysis of the bacterial 16S rRNA gene

The full-length bacterial 16S rRNA gene was amplified from one representative sample of each of the five distinct origins (ambient water, sediment, and a mucus sample from each of the three coral species) using the primers 27F (5’-AGAGTTTGATCCTGGCTCAG-3’) and 1492R (5’-GGTTACCTTGTTACGACTT-3’) (adapted from [28]). One bacterial PCR reaction (50 μL volume) consisted of 1x Dream taq buffer (Fermentas), 2 mM MgCl_2_, 0.2 mM (each) dNTP, 0.2 mg mL^-1^ BSA, 0.25 μM of each primer, 1.5 U of Dream taq polymerase (Fermentas) and 2 μL of template DNA. Thermocycling comprised an initial denaturation at 95°C for 4min; 32 cycles of amplification, at 95°C for 60 sec, 55°C for 40 sec and 72°C for 90 sec. A final extension step was done at 72°C for 15 min before holding reaction at 4°C. All PCR products were checked on 1.5% agarose gels after staining with SYBRGold (Molecular Probes, Invitrogen). For each sample, triplicate PCR reactions were pooled and purified using the PCR-Extract Mini Kit (5-Prime) and cloned with the TOPO-TA cloning kit (Invitrogen) following manufacturer’s instructions. Transformants were selected on Luria-Bertani agar plates (plus 50 μg mL^-1^ amplicilin) with X-gal (5-bromo-4-chloro-3-indolyl-β-D-galactopyranoside). White colonies were picked into 96-well plates and a PCR was run with primers M13F (5’-GTAAAACGACGGCCAG-3’) and M13R (5’-CAGGAAACAGCTATGAC-3’) to check for insert size. PCR purification and sequencing were performed by Macrogen Europe using the 27F and 1492R primers. Bacterial 16S rDNA forward and reverse sequences were assembled with CodonCode Aligner (CodonCode Corporation). Sequence information generated was deposited in GenBank under accession numbers KU243153 to KU243335. Taxonomic affiliations of the bacterial 16S rRNA gene sequences were determined using RDP Naive Bayesian rRNA Classifier [29] applying a confidence threshold of 80%. Sequences were clustered into operational taxonomic units (seqOTUs) at 98% similarity level. Rarefaction analyses as well as diversity estimates (Shannon diversity, Chao1 index) were conducted in MOTHUR v.1.23.1.

### Terminal-restriction fragment length polymorphism (T-RFLP)

For community profiling by T-RFLP, archaeal and bacterial 16S rRNA genes were amplified from extracted DNA using specific primer pairs. Fluorescent FAM- and VIC-labels were linked to the 5’-end of forward and reverse primers, respectively. Bacterial PCR reactions using the primer pair 27F-1492R followed the same procedure as described above for cloning. Archaeal 16S rRNA genes were amplified with primers 21F (5’-TTCCGGTTGATCCYGCCGGA-3’) [30] and 915R (5’-GTGCTCCCCCGCCAATTCCT-3’) [31]. Archaeal PCR resembled that for Bacteria except that 6–8 μL of template DNA was used. A touchdown PCR protocol was applied as follows: one denaturation step at 95°C for 4 min; 20 cycles of amplification at 95°C for 1 min, 65°C-55°C for 45 sec (annealing temperature decreased by 0.5°C per cycle) and 72°C for 90 sec, 20 amplification cycles at 95°C for 1 min, 55°C for 45 sec and 72°C for 1 min followed by a final extension step at 72°C for 10 min before holding reaction at 4°C.

For each sample, triplicate PCR reactions were pooled and purified using the PCR-Extract Mini Kit (5-Prime). FAM- and VIC-labeled PCR products were further digested at 37°C for 720 min. Each digest contained 200 ng of cleaned PCR product, 4 U of *Hha*I restriction enzyme (New England BioLabs), and respective buffer to a final volume of 20 μL. The reaction was terminated at 65°C for 20 min and cooled down to 4°C. The product of the restriction digest (1.5 μL) was added to 10 μL of deionized formamide and denatured at 95°C for 3 min. Each sample contained additionally 0.4 μL 1200-LIZ size marker (GeneScan) for length determination of FAM- and VIC-labelled fragments. Fragments were separated and detected in a 3130xL Genetic Analyzer (Applied Biosystems). Output electropherograms were visualized with PeakScanner v.1.0 (Applied Biosystems) and analyzed with GelCompar (Applied Maths). Electropherograms were compared to one another using the size marker as reference. To avoid scoring primers and restriction fragments larger than the size marker, peaks smaller than 50 bp and larger than 1200 bp were eliminated from the dataset. Background noise was removed after being calculated by the Wiener cut-off scale. Peak scoring was applied automatically by setting a threshold of minimum profiling at 3% and 0.4% of maximum peak height for archaeal and bacterial fingerprints, respectively, and a shoulder sensitivity of 2. Thus, the total number of OTUs output by the GelCompar software is in the same range as that obtained from PeakScanner visualizations. Low quality fingerprints were excluded from subsequent analysis. Fragment length classes were automatically assigned to existing peaks with the minimum distance between classes set to 1 bp. Peaks were binned *in silico* up to a maximum distance of 3 bp from generated classes. Output data consisted of a presence/absence matrix of all OTUs in all samples.

Additionally, T-RFLP analysis of all clones containing plasmids with amplified bacterial 16S rDNA was carried out in the same way as for the field samples, except that only 100 ng of purified PCR product were used. To validate the T-RFLP database, observed restriction fragment lengths were compared with those determined by *in silico* digestions performed in Geneious Pro 5.6.5 (http://www.geneious.com) on cloned 16S rRNA sequences using the recognition sequence of the *Hha*I restriction enzyme (http://rebase.neb.com/cgi-bin/reb_get.pl). A fingerprint map was constructed by assigning the observed peak to a taxon using the lowest possible taxonomic level and by removing all redundant peak information. A peak (i.e., a fragment of a certain length) was considered diagnostic (for a certain taxon) whenever more than 75% of the clones producing that peak belonged to the same taxon.

### Data analysis and statistics

Observed and expected T-RFLP fragment lengths obtained from the bacterial 16S rDNA clone library were compared (for FAM and VIC separately) by linear regression. The congruence of cloning and T-RFLP signatures was determined by calculating the proportion of invariable taxa (i.e., those systematically yielding the same fragment length) within each taxonomic level as well as the coefficient of variation (CV) for the length of the dominant fragments obtained from the clones assigned to each taxon at each particular taxonomic level. Rare taxa (for which only one clone was available in the library) were excluded from this analysis. The percentage identity matrix between all sequence pairs in the bacterial 16S rDNA clone library was compared by a Mantel test with 999 matrix permutations (based on Spearman’s rank correlation coefficient) to Jaccard similarity matrices (for FAM and VIC separately). The sequence pairs of the bacterial clone library were computed in Geneious using alignments produced with MUSCLE. The Jaccard similarity matrices were generated from the presence/absence data obtained after running the T-RFLP peak-scoring pipeline on processed clones.

OTU numbers (richness) retrieved from presence/absence matrices were log transformed and singletons removed (i.e., OTUs only scored in a single sample). Total numbers of bacterial and archaeal OTUs per sample were compared by applying multiple linear regression using sample origin (ambient water, sediment, *O*. *annularis*, *P*. *astreoides* and *S*. *siderea*), site (Buoy One, Snake Bay and Vaersen Bay), depth (5 m and 15 m) and patch (West, Centre and East, nested within site location) as explanatory variables and a forward and backward model selection based on the Akaike Information Criteria. Significance levels were calculated by nested-model validation approach. Posthoc pairwise comparisons were computed by Tukey’s test using a single explanatory variable at a time.

Community assembly structure was visualized in 2-dimensional Non-metric Multi-Dimensional Scaling (NMDS) plots based on Raup-Crick resemblance between samples. Raup-Crick applies a probabilistic null-modeling approach to control for differences in alpha-diversity between samples [23]. Analysis of Similarity (ANOSIM) was applied to test for significant differences between sample groupings. In addition, significant differences in beta-diversity were further addressed by applying permutational multivariate analysis of variance (PERMANOVA, with 999 permutations, using Raup-Crick similarity) after verifying the assumption of homogeneity of multivariate dispersion (diversification) using a resemblance-based permutation test (PERMDISP) [32]. Only explanatory factors yielding homogeneous multivariate dispersion were included in PERMANOVA.

The explanatory power of environmental variables on the observed community assembly was determined by Canonical Correspondence Analysis (CCA). This allowed detecting relationships between OTUs and environmental patterns. Community composition data were Hellinger-transformed prior to CCA to make it suitable for analysis by linear methods, a transformation commonly used for community composition data containing many zeros [33]. Using this transformation is based on the biological assumption that all OTUs present above a certain abundance threshold are of potential importance to the holobiont. Applying an ANOVA-like permutation test based on 999 permutations tested the significance of CCA results. The same Hellinger-transformed response data were subjected to Similarity Percentage analysis (SIMPER) after Bray-Curtis distance calculation to discriminate the OTUs responsible for most of the dissimilarity between sample groupings.

Significance of correlation between spatial (geographical) distances and community similarity (applying Raup-Crick resemblance between samples) was evaluated by Mantel test (based on Spearman’s rank correlation coefficient) with 999 matrix permutations. Geographical distance effects were also tested on individual sampling depths and for each location separately.

Alternative similarity indexes were also used. In order to evaluate the influence of OTU commonness and rarity on community assembly statistics, analyses were repeated for the complete dataset including singletons as well as for the restricted dataset including only common OTUs. Common OTUs were classified as those being present in more samples than the threshold given by the average plus one SD of the number of samples for which an OTU was detected.

All analyses were performed in Brodgar v.2.7.4 (Highland Statistics), R v.3.0.2 (vegan package) and Primer 6 v.6.1.7 (Primer-E). All tests were performed at a significance level of 0.01 and p-values adjusted when needed according to Bonferroni correction.

## Results

### Phylogenetic affiliation of mucus-associated bacteria

A total of 189 bacterial 16S rRNA gene sequences were obtained for the five clone libraries (see Table 1 for details). From these, 157 seqOTUs were resolved at the 98% similarity threshold. Rarefaction analysis showed that the sequencing effort was not sufficient to cover the expected seqOTU diversity within each habitat level (S1 Fig) as the observed diversity was only about 33% of the expected diversity (419 seqOTUs, Chao1 index).

**Table 1:** Summary of the bacterial 16S rRNA gene cloning approach. Number of clones sequenced, total and unique seqOTUs found (≥ 98% identity), richness and diversity estimates for each library. Each library represents a habitat of origin (Seawater, Sediment, *Orbicella annularis*, *Siderastrea siderea* and *Porites astreoides*).

Cloning	Number of	Total	Unique	Shannon	Chao1
library	clones	seqOTU	seqOTU	diversity	richness
Seawater	37	27	22	3.3	58
Sediment	53	50	49	3.9	285
*O*. *annularis*	45	39	33	3.7	110
*S*. *siderea*	43	35	31	3.6	58
*P*. *astreoides*	11	11	11	2.4	16

Obtained seqOTUs affiliated with bacterial sequences from 9 phyla (S2 Fig). Proteobacteria was the most abundant phylum in mucus and ambient water comprising 54% and 73% of all the sequences, respectively. Only in sediments, Cyanobacteria/chloroplasts were dominating (39%) over Proteobacteria (28%). Within the Proteobacteria, the most abundant class in mucus clone libraries was Gammaproteobacteria (44%-86%) followed by Alphaproteobacteria (14%-33%). For the ambient water, the most abundant Proteobacteria class was Alphaproteobacteria (60%) followed by Gammaproteobacteria (32%). In sediment bacteria, Gammaproteobacteria dominated (69%) followed by Deltaproteobacteria (31%). Other important phyla in the bacterial clone libraries were Bacteroidetes, Firmicutes, Actinobacteria and Acidobacteria. Overall, the proteobacterial genera *Martelella* (7%), *Vibrio* (5%), *Orientia* (4%), *Pseudoalteromonas* (3%), *Oleiphilus* (3%), *Endozoicomonas* (2%) and *Alteromonas* (2%) were among the most abundant recovered sequences.

### Linking T-RFLP patterns to bacterial 16S rRNA gene sequences

Of the 189 bacterial clones successfully sequenced, 6 and 20, for FAM and VIC, respectively, did not produce T-RFLP information, whereas 5 and 8, for FAM and VIC, respectively, showed low base assignment quality or did not yield a digest output after *in silico* digestion. These were excluded from further analyses. Thus, informative T-RFLP dataset included 180 and 163 clones from the FAM and VIC library, respectively. While some clones yielded one single T-RFLP peak (51% of clones for FAM, 31% for VIC), most clones generated smaller peaks aside a clearly dominant one. A strong and statistically significant relation was found between the observed length of the dominant restriction fragment and the expected fragment length according to *in silico* predictions (F_(1,178)_ = 6033.4, p<0.01, R^2^ = 97.1% for FAM; F_(1,161)_ = 414.39, p<0.01, R^2^ = 71.9%, for VIC; see S3 Fig). Of all the peaks obtained from the clone libraries, 38 (out of 45) FAM peaks and 10 (out of 16) VIC peaks were diagnostic for a single taxon (see S4 Fig). Clones assigned to the same taxon could, however, yield dominant peaks at different fragment length.

T-RFLP patterns became particularly consistent at and below family level (see S5 Fig). Average CV of fragment length decreased as taxonomic resolution increased. The inverse pattern was detected for the proportion of invariable taxa (S5 Fig). The population of clones belonging to the same phylum mostly yielded distinct T-RFLP peaks. In contrast, at the genus level about half of the genera consistently yielded the same dominant peak. Sequence similarity between clones was positively correlated with Jaccard similarity calculated from presence/absence peak data (Rho = 0.239, p<0.01 for FAM; Rho = 0.193, p<0.01 for VIC).

### Prokaryotic community analysis

Success of PCR amplification of 16S rRNA gene differed between Archaea and Bacteria. The archaeal 16S was amplified for only 81% of all samples even after multiple rounds of optimization: 80% of the mucus samples of *O*. *annularis*, 88% in *P*. *astreoides*, 66% in *S*. *siderea* and 100% for the sediment and ambient water samples. The bacterial 16S was successfully amplified in all samples. The number of samples used for bacterial and archaeal community analyses is shown in Table 2. The larger number of diagnostic peaks found for the FAM when compared to the VIC database (38 *versus* 10, respectively.), the higher correlation values between observed and predicted fragment length (97.1% *versus* 71.9%), as well as the higher correlation between bacterial clone sequence identity and similarity in community structure (23.9% *versus* 19.3%), all suggest that the FAM (representing the 5’-end terminal restriction fragments of 16S gene) had a higher taxonomic resolution. Thus, in further analyses we focus on results obtained for FAM. Nevertheless, a Mantel test on Bray-Curtis matrices calculated from Hellinger-transformed data showed that the FAM and VIC databases are strongly and positively correlated for both the archaeal (Rho = 0.474 p<0.01) and the bacterial communities (Rho = 0.341, p<0.01). A similar result was obtained when using Raup-Crick similarity matrices.

**Table 2:** Number of samples used for community profiling analysis and respective number of operational taxonomic units (OTUs) retrieved for the bacterial and archaeal communities analyzed. OTU numbers are given for three distinct databases (total, without singletons and only including common OTUs). Results are depicted for each of the primers used for microbial profiling based on the 16S rRNA gene.

Life	Fluorescent	Number of	Total OTU	OTU number	Common
domain	label	samples	number	(no singletons)	OTUs
Bacteria	27F-FAM	174	136	107	19
	1492R-VIC	180	157	93	20
Archaea	21F-FAM	156	64	37	8
	915R-VIC	156	58	42	6

**Fig 2:**
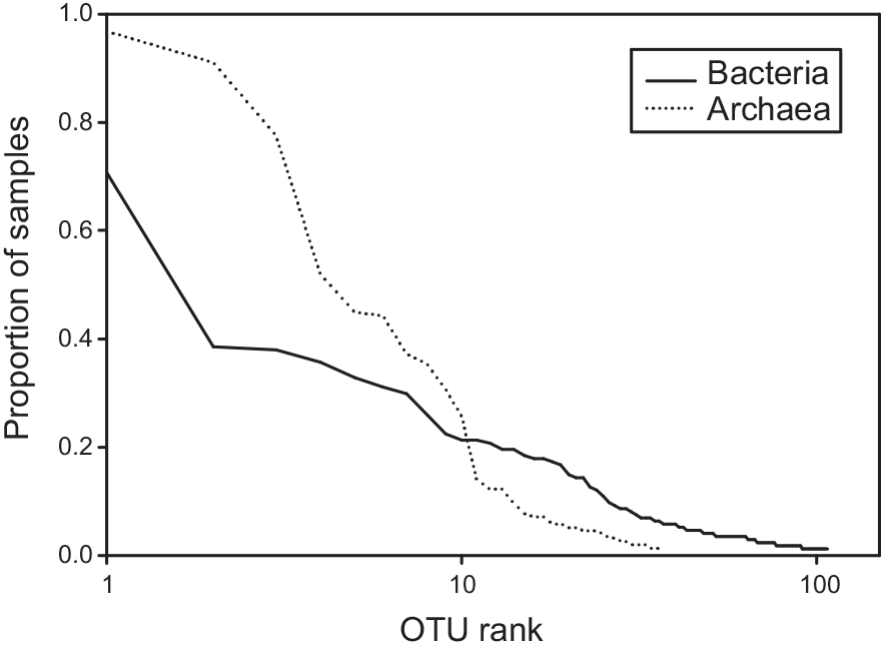
Rank frequency distribution of operational taxonomic units (OTUs) scored for the bacterial and archaeal communities analyzed. Proportion of samples represents the relative number of samples for which an OTU was detected using community profiling. Note log scale on abscissas representing the 107 and 37 OTUs identified, respectively, for the bacterial and archaeal communities.

After removing FAM singletons, a pool of 107 and 37 OTUs of Bacteria and Archaea, respectively, was obtained (see Table 2). The OTU rank frequency distributions (Fig 2) indicate that the archaeal communities were predominantly composed of either common or rare OTUs while the bacterial OTUs were more evenly distributed.

**Fig 3:**
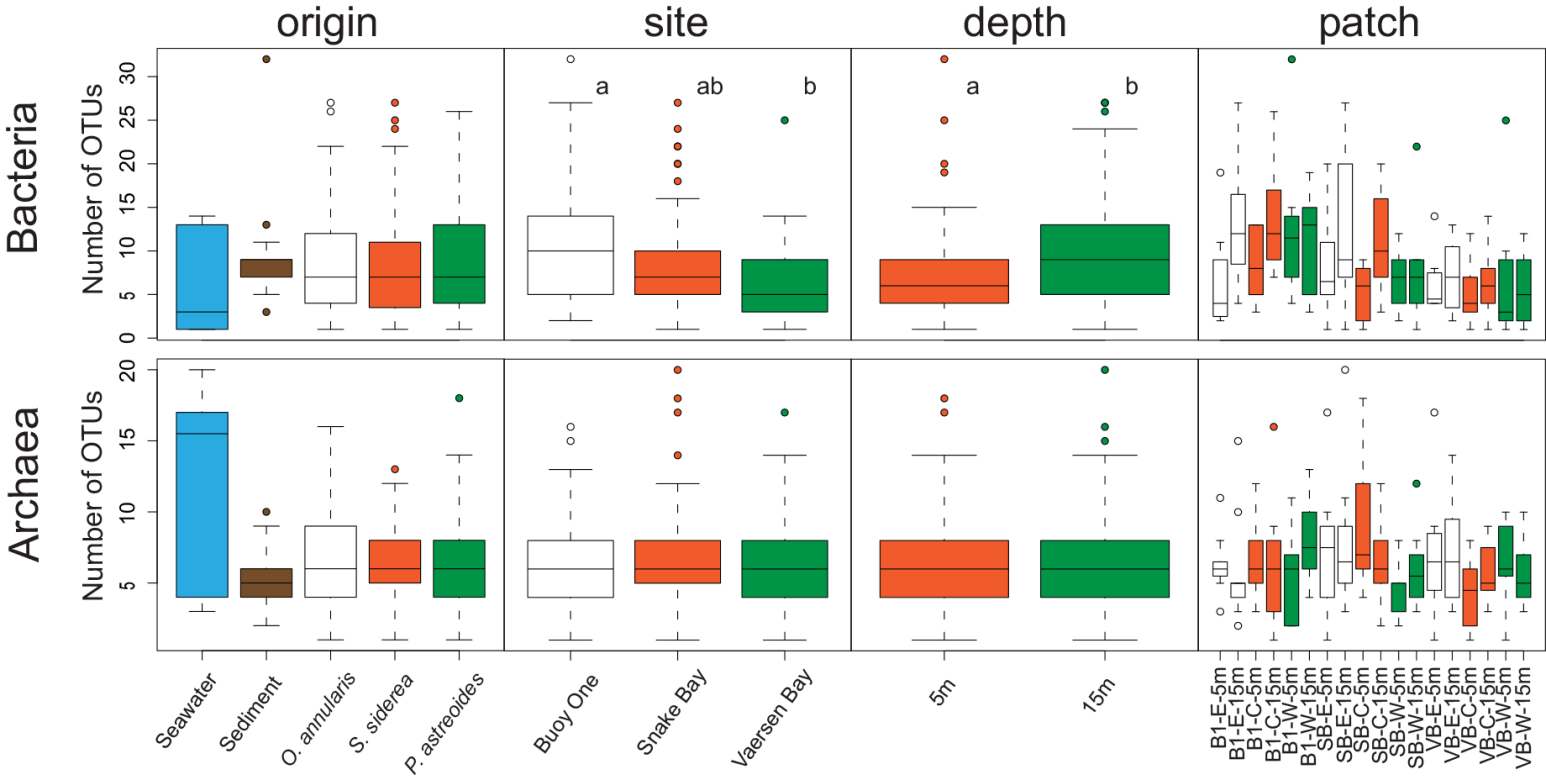
Richness in operational taxonomic units (OTUs) for bacterial and archaeal communities analyzed. OTU numbers are given according to habitat of origin (Seawater, Sediment, *Orbicella annularis*, *Siderastrea siderea* and *Porites astreoides*), site location (Buoy One, Snake Bay and Vaersen Bay), depth (5 and 15m depth) and patch location within each site-depth (West, Centre and East). Letters next to boxplots represent statistical significances determined by pairwise comparisons within each factor.

The number of bacterial OTUs per sample ranged between 1–32 while the number of archaeal OTUs varied between 1–20 (Fig 3). Average bacterial OTU numbers obtained for FAM changed significantly with depth (F_(1,166)_ = 10.05, p<0.01) and site (F_(1,166)_ = 10.139, p<0.01) with higher numbers of bacterial OTUs found at 15m than 5m depth, and at Buoy One than at Vaersen Bay (Fig 3). In contrast, average archaeal OTU numbers were not significantly affected by any of the explanatory factors. Habitat of origin (sediment, ambient water or mucus), and patch location were negligible factors for the number of bacterial or archaeal OTUs (p>0.05 for all comparisons, Fig 3).

Of all 107 bacterial OTUs obtained for the FAM dataset (S6 Fig), 63 OTUs (59%) were strictly associated with coral mucus and only 1 OTU was exclusively found in the sediment and ambient water. From the 106 bacterial OTUs associated with mucus, 47 OTUs (44%) were shared by all three coral species and 43 OTUs (41%) were shared between mucus and the environment, with most of these OTUs occurring in sediments. This distribution pattern was consistent for all coral host species (data not shown).

For Archaea, only 1 out of 37 OTUs was unique to the surrounding environment (S6 Fig) while 7 OTUs (19%) were strictly associated with coral mucus and 29 OTUs (78%) were shared between mucus and the sediment or (preferentially) ambient water. This pattern was consistent for all host species (data not shown). Out of the 36 archaeal OTUs associated with mucus, most of them (58%) were shared between all three coral species.

**Fig 4:**
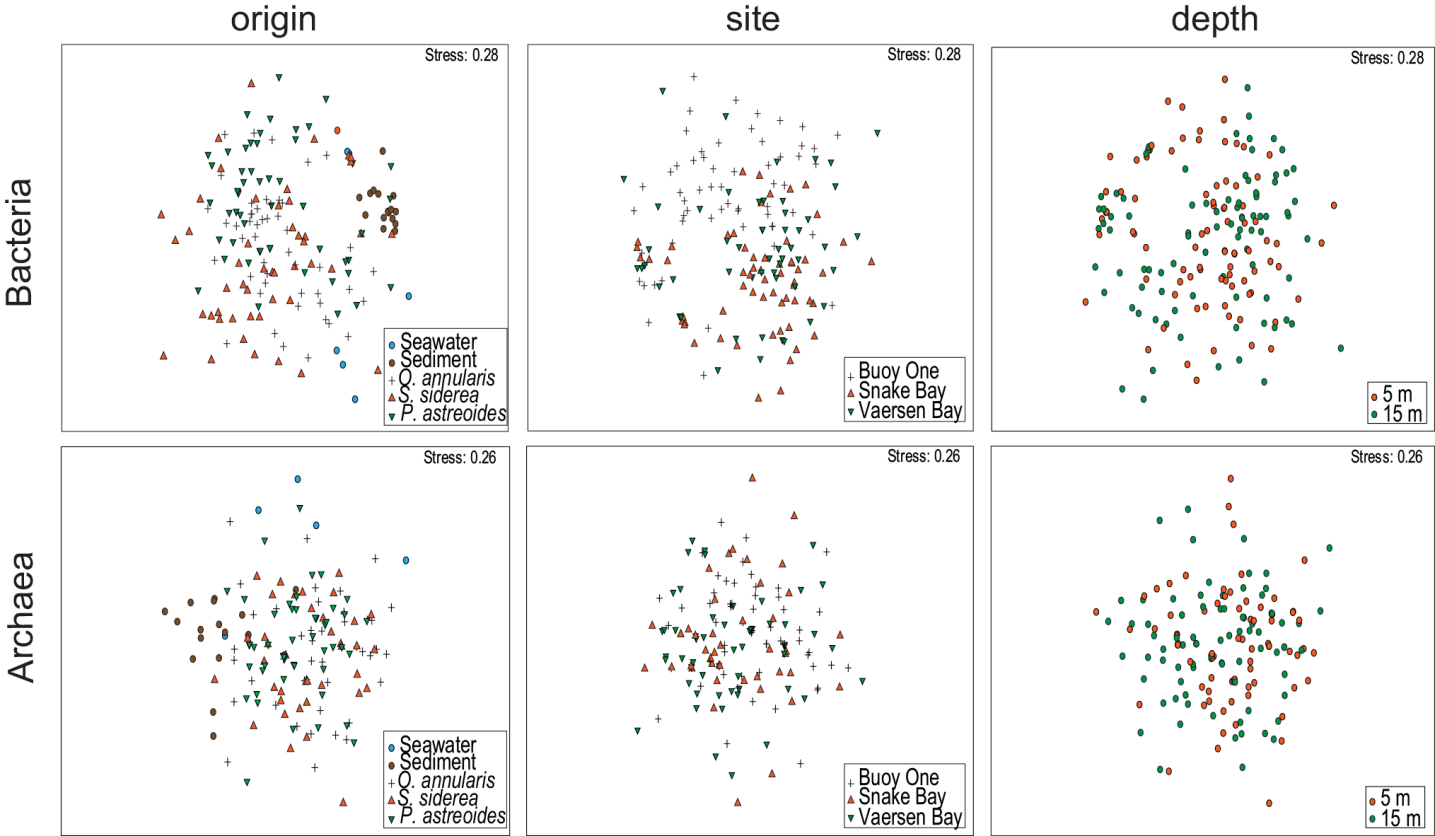
Community structure of bacterial and archaeal communities analyzed. Two-dimensional Non-metric Multi-Dimensional Scaling (NMDS) ordination depicting variation in bacterial and archaeal community structures according to habitat of origin (Seawater, Sediment, *Orbicella annularis*, *Siderastrea siderea* and *Porites astreoides*), site location (Buoy One, Snake Bay and Vaersen Bay) and depth (5 and 15m depth).

Variation in bacterial community structure visualized in 2-dimensional NMDS (Fig 4) showed that the bacterial communities clustered based on sample origin. Sediment and ambient water bacterial communities were clearly separated from the bulk bacterial community inhabiting mucus (ANOSIM R = 0.436, p<0.01). Mucus-associated bacterial communities of the same coral species were significantly more similar than those found in different coral species (ANOSIM R = 0.15, p<0.01), although there was a considerable overlap between communities belonging to different species, as seen in Fig 4. Overall, bacterial communities were structured according to site (ANOSIM R = 0.105, p<0.01) but not to depth (ANOSIM R = 0.005, p>0.05). These same patterns were also found for the mucus community alone (ANOSIM R = 0.151, p<0.01, for site; ANOSIM R = 0.015, p>0.05, for depth), but no site or depth effect in community structuring were found when analysis was restricted to sediment and ambient water (ANOSIM, p>0.05 for both). Patch location within site was a negligible factor to the structuring of the bacterial community (ANOSIM, p>0.05 for all comparisons).

Archaeal community structure (Fig 4) differed among habitat of origin (ANOSIM R = 0.416, p<0.01), with sediment and ambient water archaeal communities clustering apart from mucus samples. No coral species-specific differences were detectable in the community composition of mucus-associated Archaea (ANOSIM R = 0.015, p>0.05). Also, the archaeal communities were not structured according to site (ANOSIM R = 0.024, p>0.01) or depth (ANOSIM R = 0.019, p>0.01). These patterns were also found when analyzing only sediment and ambient water archaeal communities (ANOSIM R = 0.07, p>0.05 for site; ANOSIM R = 0.01, p>0.05 for depth). Mucus-associated archaeal communities, however, showed differences with depth (ANOSIM R = 0.035, p<0.01). Patch location (overall or within each site) had no influence on archaeal community structure (ANOSIM, p>0.05 for all comparisons).

A homogeneous diversification (multivariate dispersion) among sampling groups (PERMDISP, p>0.01, S7 Fig) was found for most environmental factors studied (host species, site and depth) and, as such, a PERMANOVA was used to investigate which of these environmental factors significantly influence community structuring of Bacteria and Archaea. For Bacteria, only site had a significant effect on community variability (pseudo F_(2,171)_ = 1.0589, p<0.01, S1 Table). These results were partially confirmed when analyzing mucus-associated bacterial communities separately (see S2 Table), for which the coral host also had an effect on bacterial community variability (pseudo F_(2,147)_ = 1.0665, p<0.01). In contrast, for Archaea, there was no effect of site or depth (or interaction effects) on community variability (p>0.05 for both statistics, see S3 Table), even not when examining only mucus archaeal communities (see S4 Table), for which the effect of host species was also non-significant.

**Fig 5:**
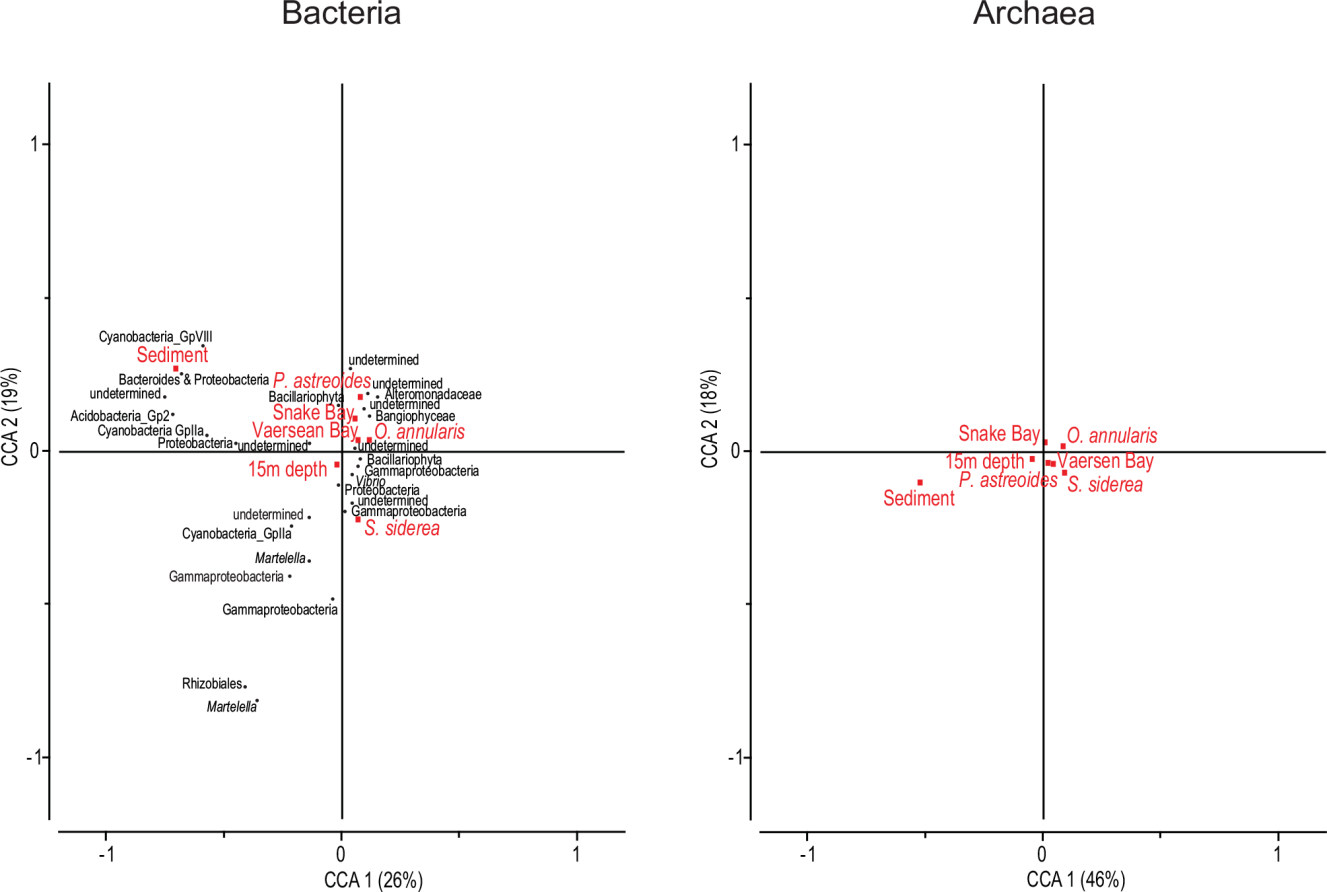
Main environmental factors driving variation in community structure for the bacterial and archaeal communities analyzed. Canonical Correspondence Analysis (CCA) depicting relationships between operational taxonomic units (OTUs) and environmental explanatory variables (habitat of origin, site location and depth) for the bacterial and archaeal communities. OTU identification not available for Archaea. Bacterial OTUs shown were discriminated by SIMPER analysis as each of them explains more than 2% of the community similarity within each sampling grouping. Note that reference level of each explanatory variable (i.e., Seawater, Buoy One and 5m depth) is represented by the intersection point of the two axes. Percentages represent amount of whole variation accounted for by each axis.

CCAs confirmed that bacterial community assembly responded to most explanatory variables studied: sample origin, site and depth (see Table 3 for significances, and widespread position of variables in Fig 5). Bacterial communities associated with sediments were the most divergent in relation to ambient water (Fig 5 and S8 Fig). Sediment bacterial community was dominated by indicator taxa within the phyla Acidobacteria, Cyanobacteria, Bacteroidetes and Proteobacteria. In the latter, Deltaproteobacteria were dominant. Ambient water bacterial communities were characterized by the recurring presence of an undetermined taxon, as well as members of the Gammaproteobacteria and Cyanobacteria. The mucus of the sampled coral species was always dominated by Gammaproteobacteria, within which the family Alteromonadaceae was characteristic. Among the mucus habitats, *O*. *annularis* harbored the most similar community to ambient seawater. *P*. *astreoides* was characterized by a dominance of Alteromonadaceae, while *S*. *siderea* was dominated by Gammaproteobacteria. All three sites were characterized by the occurrence of Gammaproteobacteria with an increasing dominance of Alteromonadaceae and diatom-affiliated chloroplasts from east (Buoy One) to west (Vaersen Bay).

**Table 3:** Summary of factor significance for Canonical Correspondence Analysis (CCA) of the bacterial and archaeal communities analyzed. CCA uses as references the conditional effects: seawater, Buoy One and 5m depth (after a test of 999 permutations) for, respectively, habitat of origin, site location and depth.

Life	Conditional	F	P
domain	effects	statistic	value
Bacteria	Sediment	4.668	0.001
	*O*. *annularis*	2.682	0.001
	*S*. *siderea*	3.390	0.001
	*P*. *astreoides*	3.279	0.001
	Snake Bay	1.926	0.001
	Vaersen Bay	2.508	0.001
	15m depth	1.591	0.002
Archaea	Sediment	6.311	0.001
	*O*. *annularis*	2.754	0.011
	*S*. *siderea*	1.246	0.168
	*P*. *astreoides*	1.148	0.250
	Snake Bay	1.435	0.055
	Vaersen Bay	1.286	0.119
	15m depth	1.475	0.024

In contrast to Bacteria, archaeal communities were less structured (Fig 5 and S8 Fig). Only depth and habitat of origin (i.e., archaeal community composition in sediment and in mucus of *O*. *annularis* compared to that of ambient water) contributed significantly to the structuring of the community (see Table 3).

A negative relationship was found between bacterial community similarity and geographical distance (Rho = -0.086, p<0.01). This distance effect was also found when testing each depth separately. Moreover, a significant negative correlation was found when using only mucus-associated bacterial communities, but not when analysis was restricted to sediment and ambient water Bacteria. In contrast, for Archaea, no relationship was found between community similarity and geographical distance (Rho = 0.000, p>0.05). Also, no correlation was found when each sampling depth or site was tested individually.

These results were also confirmed when using Bray-Curtis similarity on Hellinger-transformed community data or Jaccard similarity on presence/absence data instead of Raup-Crick similarity. Analyses applied to the complete database containing all OTUs (including singletons) and to the restricted database including solely common OTUs yielded the same general patterns as those described above.

## Discussion

In this study, we used microbial community profiling, partially coupled to clone library sequencing, to determine differences in spatial distribution and host-specificity between bacterial and archaeal communities associated with the surface mucus layer of corals.

### Taxonomic composition of bacterial and archaeal communities

The mucus-inhabiting bacterial community consists mainly of members of the class Gammaproteobacteria, particularly of the family Alteromonadaceae. These are known to establish tight associations with reef-building corals [1, 6]. Recently, Gammaproteobacteria of the genus Endozoicomonas have been shown to dominate the tissue microbiome of the coral *Stylophora pistillata* [34]. In our clone libraries, we also identified Endozoicomonas-related sequences recovered from the mucus of *Porites astreoides*. Another recent study has shown that *Roseobacter*, *Alteromonas* and *Vibrio* species are the most abundant taxa released by planulating *Pocillopora damicornis* colonies supporting the notion that their presence may be advantageous to the coral host [35]. In our study, *Vibrio*-related sequences were found in *P*. *astreoides* and *O*. *annularis*, *Alteromonas* was recovered from the mucus of *S*. *siderea* as well as from the sediment, whereas *Roseobacter* was not found. Overall, the mucus-associated bacterial community consists of members often associated with a copiotrophic lifestyle (e.g., *Vibrio*, *Alteromonas*), while in the ambient water Cyanobacteria and members of the order Rhizobiales dominated the bacterial community.

Our attempts to establish clone libraries for Archaea were not successful and hence, information on the phylogenetic composition of the archaeal community is lacking in this study. Previous studies, however, using the same primer pair (21F-915R) as we used for the archaeal T-RFLP approach, have retrieved representatives from both the Thaumarchaeota (former Crenarchaeota Marine Group I.1 assigned sequences) and Euryarchaeota [7, 11]. A metagenomic approach has recently shown that Thaumarchaeota are more abundant in sandy sediments than in coral reef waters [12]. While the studies of Wegley et al. [7] and Siboni et al. [11] on archaeal diversity were performed at the tissue level and reported a higher abundance of Thaumarchaeota than Euryarchaeota, Kellogg [2] studying coral surface mucus reported that Euryarchaeota are more abundant than Thaumarchaeota.

### Microbial community profiling

Fingerprinting techniques such as T-RFLP have been applied in the past to describe bacterial assemblages in coral reef sediments [36] and to study the interspecific and spatial variation of coral-associated bacteria [18], and are still an alternative to state-of-the-art high-throughput sequencing methods when large sample sets need to be analyzed (to ensure proper replication and statistical power) and budgets are low [37]. Unlike next generation sequencing, which allows a deeper characterization of the community (see, e.g., [6]), the T-RFLP approach only resolves the most abundant microbial taxa, present at >0.1% of total community DNA [38]. Thus, the detection level of the T-RFLP approach does not resolve the rare component of the coral reef microbiome (sensu [39]). However, several recent studies comparing the two methods have shown that, regarding community structure and dynamics of the community, the same conclusions can be inferred from both approaches [40, 41]. Furthermore, our conservative approach using only the presence/absence of peaks in the electropherogram rather than peak height or area minimizes the potential impact of PCR biases but does not allow distinguishing the relative contribution of distinct OTUs for each individual sample. Nevertheless, the Hellinger transformation applied allows generating quantitative data representative of the relative dominance of the identified OTUs [42].

Linking 16S rRNA gene sequence information obtained from clone libraries to the T-RFLP database [43] showed high reproducibility (with more than 97% of the variability in fragment length being explained by the *in silico* database, S3 Fig) and recovered significant taxonomic information contained in the community. Community profiling produced similar profiles (characterized by the CV of average fragment length and the proportion of invariable taxa) whenever closely related taxa were compared (S5 Fig), but this tendency was not pervasive under the family taxonomic level (which resolved peak length variability in a similar way to the genus level). In any case, the number of diagnostic peaks would probably have been further reduced under a higher sequencing effort of the clone libraries, since the chance of finding peak length redundancy would increase, particularly considering that the expected number of unseen seqOTUs is rather high when compared to the observed richness, and that the rarefaction curves are far from reaching a plateau.

### Community structure among coral reef habitats and host species

The prokaryotic community off the southern coast of Curaçao is highly structured with distinct bacterial and archaeal communities present in ambient water, sediment and in coral surface mucus (see Fig 5 and S8 Fig). These habitat-related differences are not attributable to differences in alpha-diversity since the resemblance index used (Raup-Crick similarity) excludes this effect. Also, no differences were found in OTU richness (alpha-diversity) between the three habitats (see Fig 3). Community structure is, however, known to be sensitive to differences in diversification (multivariate dispersion) among groups.

Differences in diversification between habitats (see S7 Fig) might be exacerbated by the varying number of samples obtained from each habitat. Nevertheless, it is likely that taxonomic shifts in the community are responsible for the differences in community structure (beta-diversity) between these habitats.

Previous studies applying clone library sequencing and community profiling techniques (T-RFLP and DGGE) suggest, like our results do, that dominant bacterial communities inhabiting corals are distinct from those occurring in the surrounding environment [1, 44]. Such a pattern is assumed to occur in Archaea as well [7] albeit not all studies provide such evidence [2]. The present, non mutually exclusive understanding of the coral microbiome is, however, that the dominant coral-associated microbes are members of the rare biosphere in the ambient water [6]. Taxonomic shifts among the studied coral reef habitats are most likely induced by the differences in organic matter composition and concentration in the mucus and sediment [45], hence are bottom-up controlled. However, examples of top-down control on mucus-associated microbial composition also exist, such as the typical behavior of shedding aged mucus layers exhibited by poritid corals [9]. Taxonomic shifts from oligotrophic to copiotrophic Bacteria have been reported from open waters to coral seawater [12]. The nutrient enriched coral mucus and superficial sediments might offer a broader range of metabolic possibilities for the microbial communities than the more oligotrophic ambient seawater.

In the present study and in others it has been shown that mucus-associated bacterial communities vary greatly between distinct host species living in the same environment [1]. However, archaeal communities apparently do not establish host species-specific associations (Figs 4 and 5), at least based on the level of resolution given by the 16S rRNA gene. Results further support the notion that the association between corals and Archaea is rather cosmopolitan [7], as opposed to the more specific associations Bacteria establish with corals.

### Spatial variation and community turnover over reefs

Site location provides a stronger axis for bacterial community differentiation than depth (5 *versus* 15m depth, Figs 4 and 5). Similar community structuring over spatial scales has been shown for both tissue- [18] and mucus-associated bacterial communities [20] while other studies report no influence of geographical location on the mucus bacterial microbiome [46]. In contrast to Bacteria, archaeal communities showed no signs of spatial structuring between sites (Figs 4 and 5, and Table 3). Thus, archaeal communities inhabiting coral reefs apparently establish rather uniform assemblages across large geographical scales [11].

Overall, we found no differences in community structure attributable to within-site patch location (west, center and east patches) or within-site geographic distance (in the range of 0–100 m) for archaeal and bacterial communities. This is in accordance with microbial biogeography studies reporting no effect of spatial distance below spatial scales of a few kilometers [47].

In one of the few studies investigating coral-associated microbial communities off Curaçao, Rodriguez-Lanetty et al. [25] concluded that there is no structuring pattern of the dominant bacterial community associating with the tissue of *Porites astreoides* (determined by DGGE) across the spatial scale. In contrast, the rare members of the bacterial community (determined by high-throughput sequencing) differed both in composition and abundance as a function of geographic location. Klaus et al. [24] used T-RFLP patterns to show that local environmental conditions can influence the distribution of Bacteria inhabiting coral tissues, however, with different trends among different coral species. The authors linked the structuring effect of the environment on bacterial communities to the amount of human pollutants, known to gradually decrease in a westerly direction from the urban area of Willemstad [26].

Our results also suggest that there is an effect of geographic distance on bacterial community composition. These patterns have been shown for the complete dataset and also for the mucus-associated bacterial community alone but not for the surrounding environment (sediment and ambient water). This suggests that geographic and spatial differences observed for the mucus-associated bacterial community are likely driven by the host, but we cannot ascertain here the potential role of environmental gradients or heterogeneity. Similar relationships between variation in community structure and geographical distance have been discussed elsewhere, and are referred to as community turnover whenever they happen along a spatial, temporal or environmental gradient [23]. In our study, the contrasting degree of community turnover found for Bacteria and Archaea in coral mucus along a east to west gradient probably reflects distinct combinations of the processes underlying microbial biogeographic patterns such as selection, drift, dispersal and mutation [22], part of which might be dependent on the physiology, adaptations and population dynamics of the hosts.

### Archaeal and bacterial communities in coral mucus

We attribute the unsuccessful amplification of the archaeal 16S rRNA gene from coral mucus samples to the low abundance of Archaea in coral mucus. A low relative abundance of Archaea was also reported for other coral reef environments [12]. Wegley et al. [7] obtained a variable archaeal 16S amplification success rate, varying from 26% to 100% of tested samples depending on the coral species sampled. Kellogg [2], however, was also unable to amplify archaeal 16S from some mucus samples, particularly from *P*. *astreoides*.

In order to exclude that the community differences found between Archaea and Bacteria are the result of the lower abundance in Archaea (and consequent decreased OTU richness retrieved), we repeated the analysis for the restricted database including only the 19 most common bacterial OTUs. This analysis corroborated the community structuring found for Bacteria with the general database, suggesting that the low degree of community turnover found for Archaea over the spatial scale studied is likely not related to its intrinsically low OTU richness.

Generally, we found striking differences between Archaea and Bacteria. Whereas the bacterial community responded to most environmental factors studied, the archaeal community did not respond to host species, site location or geographic distance. Kellogg [2] concluded that the majority of archaeal sequences within the surface mucus of three Caribbean reef-building corals is derived from the ambient water. In our study, we found that while only 41% of the bacterial OTUs are shared between mucus and the environment, 78% of all archaeal OTUs are shared, with most mucus archaeal OTUs co-occurring in the three host species (S6 Fig). These findings support the perspective that whereas Bacteria form a specific and integral part of the coral mucus microbiome, Archaea are part of its rare microbiome and/or constitute visitors originating from the surrounding environment. Coral mucus is known to trap particles and microbes transported by the water column [45], indicating that many microbes present in coral mucus may also be commensal forms, neither beneficial nor harmful to the coral species [3]. Archaea would then, unlike Bacteria, not be establishing specific associations with the coral host [7] but rather, taking advantage of non host-specific niches. Such capacity could relate to metabolic capabilities known for Archaea. Siboni et al. (2008) proposed a model in which distinct mucus associated Archaea perform either ammonia oxidation or denitrification depending on oxic *versus* anoxic conditions within the mucus layer. In contrast, Bacteria might occupy more consolidated niches allowing them more close interactions with their host [14]. Our findings suggest that mucus-associated Archaea and Bacteria differ in community turnover among reefs and host-specificity. Whether such differences match a distinct vertical distribution pattern of Archaea and Bacteria within the thin coral mucus layer warrants further investigation.

## Supporting Information

S1 Fig Rarefaction curves for the different 16S rRNA gene clone libraries generated. Each library represents a habitat of origin (Seawater, Sediment, *Orbicella annularis*, *Siderastrea siderea* and *Porites astreoides*). Level of diversity detected changed as a function of sequencing effort without reaching a plateau, showing the coverage was rather low for all libraries.
(EPS)

S2 Fig Bacterial community composition resolved by the 16S rRNA gene clone libraries. Each library represents a habitat of origin (Seawater, Sediment, *Orbicella annularis*, *Siderastrea siderea* and *Porites astreoides*). Colour codes used for each taxonomic level represented in the libraries are given. Taxonomic affiliation shown is at the phylum level except for the Proteobacteria (classes are given). Note ordinates are normalized to total number of clones for each library.
(EPS)

S3 Fig Validation of the microbial community profiling approach applied. Observed *versus* expected terminal-restriction fragment lengths for the 5’ end (FAM) and the 3’ end (VIC) of the bacterial 16S rRNA gene obtained after coupling clone libraries to community profiling. Diagonal line represents equal fragment lengths between *in silico* digestions of sequences obtained from clone libraries and terminal-restriction length polymorphism analysis ran for those same clones.
(EPS)

S4 Fig Community profiling fingerprint maps. These represent diagnostic peaks for the 5’ end (FAM) and the 3’ end (VIC) of the bacterial 16S rRNA gene obtained after coupling clone libraries to community profiling. Fragment length is given between brackets next to respective peak. Bacterial affiliation was resolved for each peak at the lowest possible taxonomic level. Phylum names are given between quotation marks. Peaks were considered diagnostic whenever generated by a population of clones of which more than 75% belong to the same taxon. This fraction is given between brackets after taxonomic affiliation.
(EPS)

S5 Fig Congruence of community profiling signatures within each taxonomic level. Graphs depict the CV of average fragment length and the proportion of invariable taxa within each taxonomic level for the 5’ end (FAM) and the 3’ end (VIC) of the bacterial 16S rRNA gene obtained after coupling clone libraries to community profiling.
(EPS)

S6 Fig Number of unique, shared and ubiquitous operational taxonomic units (OTUs) found for the bacterial and archaeal communities analyzed. Venn diagrams on the left represent OTU numbers scored for the studied habitats of origin (Sediment, Seawater and Mucus). Venn diagrams on the right represent mucus-associated OTUs and their distribution over the three studied coral species (*Orbicella annularis*, *Siderastrea siderea* and *Porites astreoides*). All data originated from community profiling (T-RFLP).
(EPS)

S7 Fig Diversification of bacterial and archaeal communities among sampling groups analyzed. Graphs depict the distance to centroid (as a measure of multivariate dispersion) for each habitat of origin (Seawater, Sediment, *Orbicella annularis*, *Siderastrea siderea* and *Porites astreoides*), site location (Buoy One, Snake Bay and Vaersen Bay), depth (5 and 15m depth) and patch location within each site-depth. Abbreviations for patch give, in this order, the site location, the patch location (W, C and E represent West, Center and East, respectively, as depicted in Fig 1) and the depth. Letters next to boxplots represent statistical significances determined by pairwise comparisons within each factor.
(EPS)

S8 Fig Relative abundance of (A) bacterial and (B) archaeal operational taxonomic units (OTUs) driving differences between sampling groups analyzed. Abundance is depicted as Hellinger-transformed data from original profiling presence/absence data. Only OTUs explaining more than 2% of the community similarity within each sampling grouping according to SIMPER analysis are given. Crosses mark OTUs not listed as main OTUs for a particular factor. Tentative taxonomic affiliation for Bacteria determined by coupling community profiling to clone library sequencing (see S4 Fig.). For the archaeal community no data is shown for variation between sites because this factor was not considered to significantly drive the community (see results of Canonical Correspondence Analysis).
(EPS)

S1 Table Environmental factors significantly contributing to community structuring of the bacterial reef community analyzed. Summary of permutational multivariate analysis of variance obtained for the bacterial community using the whole terminal-restriction fragment length polymorphism dataset.
(DOCX)

S2 Table Environmental factors significantly contributing to community structuring of the bacterial community associated with coral mucus. Summary of permutational multivariate analysis of variance obtained for the bacterial community using mucus samples only (sediment and seawater samples are excluded).
(DOCX)

S3 Table Environmental factors significantly contributing to community structuring of the archaeal reef community analyzed. Summary of permutational multivariate analysis of variance obtained for the archaeal community using the whole terminal-restriction fragment length polymorphism dataset.
(DOCX)

S4 Table Environmental factors significantly contributing to community structuring of the archaeal community associated with coral mucus. Summary of permutational multivariate analysis of variance obtained for the archaeal community using mucus samples only (sediment and seawater samples are excluded).
(DOCX)
