# Supplementary material for: Archaeal and Bacterial Communities Associated with the Surface Mucus of Caribbean Corals Differ in Their Degree of Host Specificity and Community Turnover Over Reefs
Source: PLoS One. 2016 Jan 20;11(1):e0144702. doi: 10.1371/journal.pone.0144702 (PMC4720286; doi:10.1371/journal.pone.0144702)
Supplement: S1 Table — Summary of permutational multivariate analysis of variance obtained for the bacterial community using the whole terminal-restriction fragment length polymorphism dataset. (DOCX) [file pone.0144702.s009.docx]

**Table S1. Environmental factors significantly contributing to community structuring of the bacterial reef community analyzed.** Summary of permutational multivariate analysis of variance obtained for the bacterial community using the whole terminal-restriction fragment length polymorphism dataset.

| **Source** | **df** | **SS** | **MS** | **Pseudo-F** | **P(perm)** | **Unique perms** |
| --- | --- | --- | --- | --- | --- | --- |
| Site | 2 | 10457 | 5228.4 | 1.0589 | 0.001 | 979 |
| Depth | 1 | 4953.1 | 4953.1 | 1.0031 | 0.503 | 980 |
| SitexDepth | 2 | 9894 | 4947 | 1.0019 | 0.486 | 971 |
| Residuals | 168 | 8.30 x 10^5^ | 4937.6 |  |  |  |
| Total | 173 | 8.55 x 10^5^ |  |  |  |  |
