# Supplementary material for: Archaeal and Bacterial Communities Associated with the Surface Mucus of Caribbean Corals Differ in Their Degree of Host Specificity and Community Turnover Over Reefs
Source: PLoS One. 2016 Jan 20;11(1):e0144702. doi: 10.1371/journal.pone.0144702 (PMC4720286; doi:10.1371/journal.pone.0144702)
Supplement: S2 Table — Summary of permutational multivariate analysis of variance obtained for the bacterial community using mucus samples only (sediment and seawater samples are excluded). (DOCX) [file pone.0144702.s010.docx]

**Table S2.** **Environmental factors significantly contributing to community structuring of the bacterial community associated with coral mucus.** Summary of permutational multivariate analysis of variance obtained for the bacterial community using mucus samples only (sediment and seawater samples are excluded)

| **Source** | **df** | **SS** | **MS** | **Pseudo-F** | **P(perm)** | **Unique**  **perms** |
| --- | --- | --- | --- | --- | --- | --- |
| Species | 2 | 10503 | 5251.7 | 1.0665 | 0.002 | 988 |
| Site | 2 | 10541 | 5270.7 | 1.0704 | 0.001 | 985 |
| Depth | 1 | 4962.4 | 4962.4 | 1.0078 | 0.473 | 992 |
| SpxSi | 4 | 19797 | 4949.3 | 1.0051 | 0.393 | 987 |
| SpxDe | 2 | 9989.7 | 4994.9 | 1.0144 | 0.323 | 985 |
| SixDe | 2 | 9903 | 4951.5 | 1.0056 | 0.461 | 989 |
| SpxSixDe | 4 | 19757 | 4939.2 | 1.0031 | 0.451 | 984 |
| Residuals | 132 | 6.50 x 10^5^ | 4924.2 |  |  |  |
| Total | 149 | 7.35 x 10^5^ |  |  |  |  |
