# Supplementary material for: Archaeal and Bacterial Communities Associated with the Surface Mucus of Caribbean Corals Differ in Their Degree of Host Specificity and Community Turnover Over Reefs
Source: PLoS One. 2016 Jan 20;11(1):e0144702. doi: 10.1371/journal.pone.0144702 (PMC4720286; doi:10.1371/journal.pone.0144702)
Supplement: S3 Table — Summary of permutational multivariate analysis of variance obtained for the archaeal community using the whole terminal-restriction fragment length polymorphism dataset. (DOCX) [file pone.0144702.s011.docx]

**Table S3. Environmental factors significantly contributing to community structuring of the archaeal reef community analyzed.** Summary of permutational multivariate analysis of variance obtained for the archaeal community using the whole terminal-restriction fragment length polymorphism dataset.

| **Source** | **df** | **SS** | **MS** | **Pseudo-F** | **P(perm)** | **Unique perms** |
| --- | --- | --- | --- | --- | --- | --- |
| Site | 2 | 9952.9 | 4976.5 | 1.0102 | 0.205 | 979 |
| Depth | 1 | 4972.6 | 4972.6 | 1.0094 | 0.365 | 988 |
| SitexDepth | 2 | 9800.4 | 4900.2 | 0.9947 | 0.724 | 977 |
| Residuals | 150 | 7.39 x 10^5^ | 4926.3 |  |  |  |
| Total | 155 | 7.64 x 10^5^ |  |  |  |  |
