# Supplementary material for: Archaeal and Bacterial Communities Associated with the Surface Mucus of Caribbean Corals Differ in Their Degree of Host Specificity and Community Turnover Over Reefs
Source: PLoS One. 2016 Jan 20;11(1):e0144702. doi: 10.1371/journal.pone.0144702 (PMC4720286; doi:10.1371/journal.pone.0144702)
Supplement: S4 Table — Summary of permutational multivariate analysis of variance obtained for the archaeal community using mucus samples only (sediment and seawater samples are excluded). (DOCX) [file pone.0144702.s012.docx]

**Table S4. Environmental factors significantly contributing to community structuring of the archaeal community associated with coral mucus.**  Summary of permutational multivariate analysis of variance obtained for the archaeal community using mucus samples only (sediment and seawater samples are excluded).

| **Source** | **df** | **SS** | **MS** | **Pseudo-F** | **P(perm)** | **Unique**  **perms** |
| --- | --- | --- | --- | --- | --- | --- |
| Species | 2 | 9874.8 | 4937.4 | 1.0034 | 0.470 | 985 |
| Site | 2 | 9918.2 | 4959.1 | 1.0078 | 0.416 | 991 |
| Depth | 1 | 4978.2 | 4978.2 | 1.0116 | 0.435 | 992 |
| SpxSite | 4 | 19657 | 4914.3 | 0.99866 | 0.547 | 984 |
| SpxDe | 2 | 9821.7 | 4910.9 | 0.99796 | 0.549 | 989 |
| SixDe | 2 | 9812 | 4906 | 0.99697 | 0.556 | 997 |
| SpxSixDe | 4 | 19713 | 4928.2 | 1.0015 | 0.496 | 984 |
| Residuals | 115 | 5.66 x 10^5^ | 4920.9 |  |  |  |
| Total | 132 | 6.50 x 10^5^ |  |  |  |  |
